# Identification of potential biomarkers in Barrett’s esophagus derived esophageal adenocarcinoma

**DOI:** 10.1038/s41598-022-17107-0

**Published:** 2023-02-09

**Authors:** Nan Yi, Hailiang Zhao, Juan He, Xike Xie, Liexin Liang, Guowen Zuo, Mingyue Xiong, Yunxiao Liang, Tingzhuang Yi

**Affiliations:** 1 Department of Gastroenterology, The People’s Hospital of Guangxi Zhuang Autonomous Region, Nanning, 530021 Guangxi P. R. China; 2 Department of Gastroenterology, The First Affiliated Hospital, Jinan University, Guangzhou, 510630 P. R. China; 3 Department of Gastroenterology, Affiliated Hospital of Youjiang Medical University for Nationalities, Baise, 533000 P. R. China; 4 Youjiang Medical University for Nationalities, Baise, 533000 Guangxi P. R. China; 5 Department of Hematology, Baise People’s Hospital, Baise, 533000 Guangxi P. R. China; 6 Department of Oncology, Affiliated Hospital of YouJiang Medical University for Nationalities, Baise, 533000 Guangxi P. R. China

**Keywords:** Cancer, Computational biology and bioinformatics

## Abstract

Almost 50% of esophageal adenocarcinoma (EAC) patients progressed from Barrett’s esophagus (BE). EAC is often diagnosed at late stages and is related to dismal prognosis. However, there are still no effective methods for stratification and therapy in BE and EAC. Two public datasets (GSE26886 and GSE37200) were analyzed to identify differentially expressed genes (DEGs) between BE and EAC. Then, a series of bioinformatics analyses were performed to explore potential biomarkers associated with BE-EAC. 27 up- and 104 down-regulated genes were observed between GSE26886 and GSE37200. The GO and KEGG enrichment analysis indicated that the DEGs were highly involved in tumorigenesis. Subsequently, Weighted Gene Co-Expression Network Analysis (WGCNA) were performed to explore the potential genes related to BE-EAC, which were validated in The Cancer Genome Atlas (TCGA) database*,* and 5 up-regulated genes (*MYO1A*, *ACE2*, *COL1A1*, *LGALS4*, and *ADRA2A*) and 3 down-regulated genes (*AADAC*, *RAB27A*, and *P2RY14*) were found in EAC. Meanwhile, *ADRA2A* and *AADAC* could contribute to EAC pathogenesis and progression. *MYO1A*, *ACE2*, *COL1A1*, *LGALS4*, *ADRA2A, AADAC*, *RAB27A*, and *P2RY14* could be potential novel diagnostic and prognostic biomarkers in BE-EAC.

## Introduction

Esophageal adenocarcinoma (EAC), the predominant subtype in the west, is one of the two main histological types of esophageal cancer, and the incidence of EAC has increased nearly six-fold over the last three decades^1,2^. Long-term exposure to the acid, bile, and other stomach contents causes great injury of the squamous esophageal epithelium and increases the risk of developing Barrett’s esophagus (BE) and later EAC^3,4^. BE is the only recognized precursor of EAC. Individuals with BE are 30–125 times more likely to develop EAC than the general population, and almost 50% of EAC patients progressed from BE^5^. Patients with BE must undergo regular endoscopic surveillance, as BE surveillance carries an improved prognosis^6^. Given the high cost of endoscopy and many patients still developing EAC during endoscopic surveillance, stratification of BE patients is indispensable. Meanwhile, EAC is often diagnosed at late stages and is related to dismal prognosis. Although tremendous progress has been made in therapy, including esophagectomy, chemotherapy, and molecular targeted drugs, the 5-year survival rate of EAC remains less than 20%^7^. Therefore, it is necessary to explore potential targets for diagnosis and therapy.

Several genes from genome-wide association studies have been identified as having impacts on the pathogenesis of BE to EAC. It is reported that *ELF3*, *KLF5*, *GATA6*, *EHF*, *TTK*, *TPX2*, and *RAD54B* are important genetic modifiers played important roles in the pathogenesis and progression of BE to EAC^1,8^. Spechler et al.^9^ noted that early *CDKN2A* (*P16*) loss or methylation and subsequent loss of *P53* in non-dysplastic BE might contribute to BE-EAC progression. In addition, Dulak et al.^10^ indicated that *SMAD4*, *ARID1A*, *PIK3CA*, *SPG20*, *TLR4*, *ELMO1*, and *DOCK2* had a significant impact on BE-EAC progression. However, there are still no effective methods for stratification and therapy in BE and EAC.

Therefore, we analyzed two public datasets to identify differentially expressed genes (DEGs) among BE and EAC. Then, Weighted Gene Co-Expression Network Analysis (WGCNA) was performed to explore the potential genes related to BE-EAC. This study aimed to screen potential genes for BE-EAC progression.

## Material and methods

### Data Retrieving and Processing

Data from Gene Expression Omnibus (GEO, http://www.ncbi.nlm.nih.gov/geo) were fulfilled the inclusion criteria below: ① publication date from 2010 to 2022; ② containing BE and EAC tissue samples; ③ sample size > 3 in each group. The exclusion criteria were: ① duplicated research; ② animal or cell experiments; ③ incomplete data; ④ patients with chemotherapy or radiation treatment. Then, the gene expression profiles of GSE26886^11^ and GSE37200^12^ were downloaded from GEO. Finally, 20 BE samples and 21 EAC samples in the GSE26886; and 31 BE samples and 15 EAC samples in the GSE37200 were included in this study. EAC without chemotherapy or radiation treatment data were obtained from The Cancer Genome Atlas (TCGA) database, including 70 EAC samples and 8 normal samples adjacent to EAC.

Batch effects were corrected using “limma” R packages^13^, and principal components analysis (PCA) was carried out. Two R packages (“GEOquery” and “limma”) were used for the analysis of DEGs. The threshold for the DEGs was set as adjusted-P value < 0.05 and |log_2_ fold change (FC) |≥ 1. Volcano plots and heat maps were drawn using R package “ggplot2” (https://ggplot2.tidyverse.org/) and “complexHeatmap”^14^. Venn diagram was performed using the jvenn tool (http://jvenn.toulouse.inra.fr/app/example.html), and the overlaps represented the intersection between the two datasets.

### Gene ontology (GO) analysis and Kyoto encyclopedia of genes and genomes (KEGG) pathway enrichment analysis

To identify the function of DEGs, GO and KEGG analysis were performed using Metascape (metascape.org) database. GO is a commonly used bioinformatics tool that supply comprehensive information on gene function of individual genomic products based on defined features and is primarily divided into three parts, molecular function (MF), biological process (BP), and cellular component (CC). KEGG is a database resource for understanding high-level biological functions and utilities^15^. We determined that results were statistically significant at a level of adjusted-P < 0.05 and false discovery rate (FDR) < 0.05. Then, histograms and chord plots were generated with R package “GOplot”.

### Weighted gene co-expression network analysis (WGCNA)

Considering that GSE26886 had larger sample size of EAC, it was used to detect modules highly correlated with EAC, and WGCNA was performed using R package “WGCNA”^16^ and carried out on all genes. The scale-free topology of the networks was assessed for various values of the β shrinkage parameter, and we chose β = 8 based on scale-free topology criterion. Finally, the dynamic tree cut algorithm was applied to the dendrogram for module identification with the mini-size of module gene numbers set as 50, and similar modules were merged following a height cutoff of 0.25. In the module-trait analysis, gene-trait significance (GS) value > 0.3 and module membership (MM) value > 0.55 were defined as a threshold^17^. Then, Venn diagram was performed to explore the trait-expression-related genes.

### Exploration of trait-expression-related genes in TCGA database

Subsequently, the expression levels of trait-expression-related genes were estimated in TCGA database, a receiver operating characteristic (ROC) curve was performed to assess the diagnostic value of the genes by “pROC” R package, and survival analysis was also performed using “survival” and “survminer” R packages.

## Statistical analysis

Statistical analysis was performed using R software (Version 4.1.0, www.r-project.org). Statistical comparisons between groups of normalized data were performed using the t-test or Mann–Whitney U-test according to the test condition, and categorical data were analyzed by the χ2 test or Fisher exact test. A difference with P < 0.05 was considered significant.

## Results

### Identification of DEGs in the EAC patients

**Figure 1:**
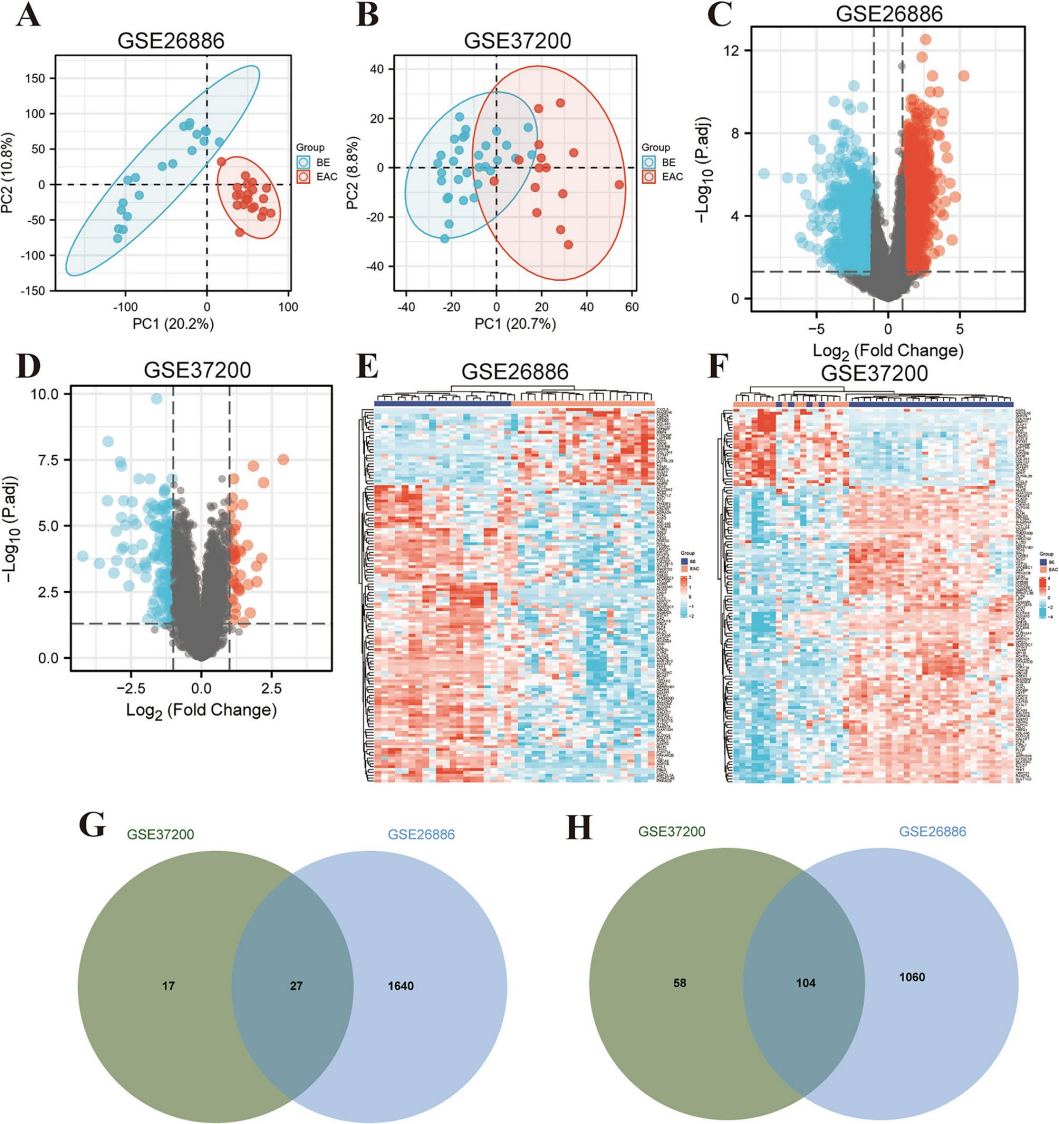
Identification of differentially expressed genes (DEGs) between BE and EAC. The principal components analysis (PCA) in GSE26886 (**A**) and GSE37200 (**B**) The volcano plot of DEGs in GSE26886 (**C**) and GSE37200 (**D**); The Heatmap of DEGs in GSE26886 (**E**) and GSE37200 (**F**); (**G**,**H**) Venn diagrams displayed the overlapping DEGs of up (**G**) and down (**H**) regulated genes between BE and EAC.

The batch effects were removed (Figure S1A,B), and the PCA showed that there were obvious differences between BE and EAC (Fig. 1A,B). the DEGs between BE and EAC in GSE26886 and GSE37200 datasets were identified, respectively (Fig. 1C,F). Then, we sought for the overlapping DEGs between the two datasets, and 27 up- and 104 down-regulated genes were observed in EAC (Fig. 1G,H).

### GO and KEGG pathway enrichment analysis

**Figure 2:**
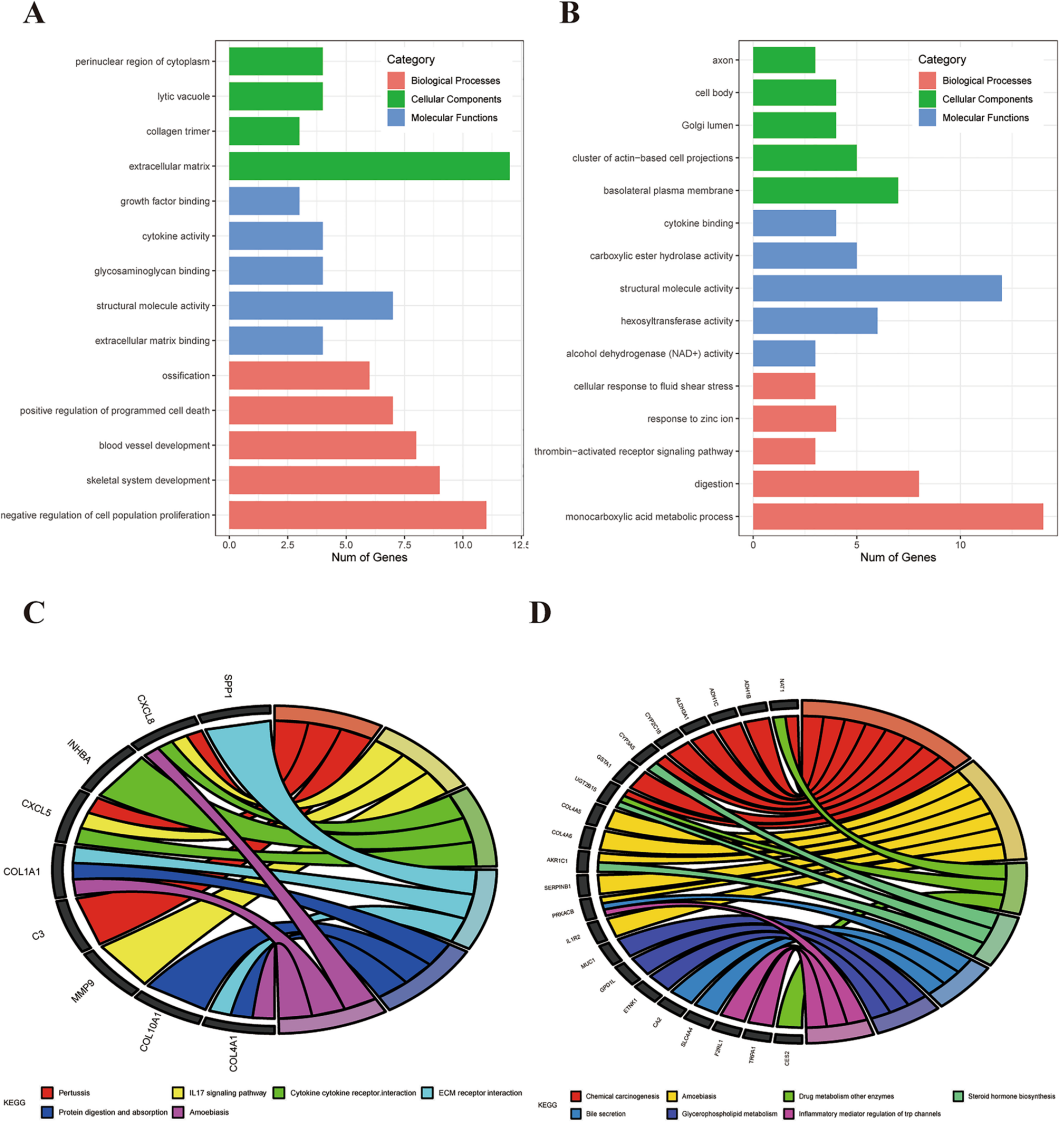
GO and KEGG pathway enrichment analysis. GO analysis of up (**A**) and down (**B**) regulated DEGs in EAC; KEGG analysis of up (**C**) and down (**D**) regulated DEGs in EAC.

To explore the potential roles of DEGs between BE and EAC, GO and KEGG pathway enrichment analyses were performed. GO analysis showed that the up-regulated genes in EAC were mainly involved in biological processes (BP) associated with negative regulation of cell population proliferation, skeletal system development, blood vessel development, positive regulation of programmed cell death, and ossification (Fig. 2A). In contrast, the down-regulated genes in EAC were mainly involved in BP associated with monocarboxylic acid metabolic process, digestion, thrombin-activated receptor signaling pathway, response to zinc ion, and cellular response to fluid shear stress (Fig. 2B). These results indicated that the DEGs were highly associated with epithelial-mesenchymal transition (EMT) and nutrition. KEGG analysis indicated that the up-regulated DEGs in EAC were primarily enriched in Pertussis, IL-17 signaling pathway, cytokine-cytokine receptor interaction, ECM-receptor interaction, protein digestion and absorption, and Amoebiasis (Fig. 2C); while the down-regulated genes in EAC were enriched in chemical carcinogenesis, Amoebiasis, drug metabolism-other enzymes, steroid hormone biosynthesis, bile secretion, glycerophospholipid metabolism, and inflammatory mediator regulation of trp channels (Fig. 2D). These results demonstrated that the DEGs were highly involved in tumorigenesis.

### Identification of key modules by WGCNA

**Figure 3:**
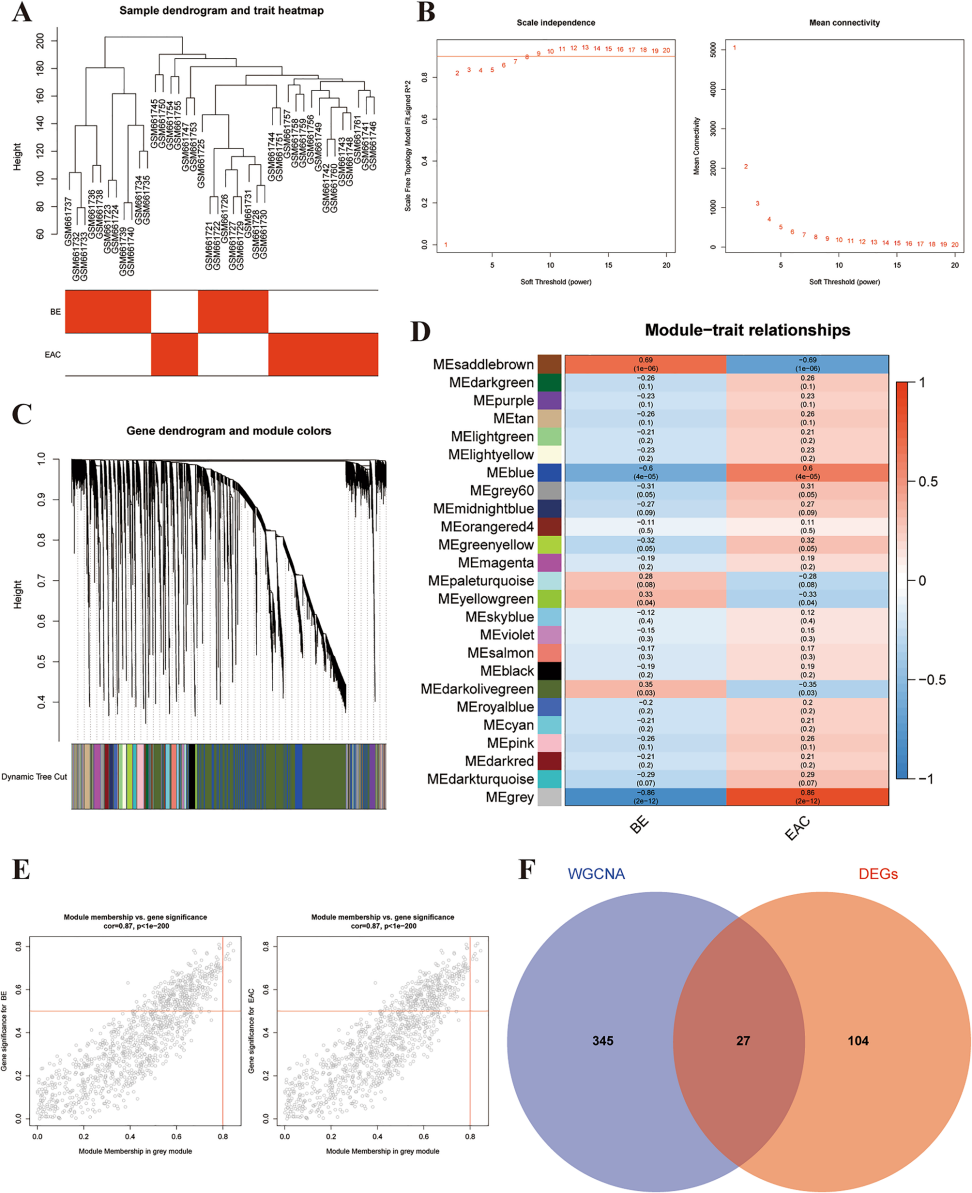
WGCNA to identify trait-related modules and genes. (**A**) Sample dendrogram and trait heat map; (**B**) calculating soft-thresholding power; Left: scale-free fit indices using different soft-thresholding powers; Right: mean connectivity using different soft-thresholding powers; (**C**) the dendrogram clustered by Dynamic Tree Cut algorithm; (**D**) the heatmap profiling the correlations between module eigengenes and the clinical characteristics; (**E**) scatter plot of gene significance in grey module; Left: BE; Right: EAC; (**F**) Venn diagrams displayed the overlapping genes between trait-related genes in grey module and DEGs.

WGCNA analysis provides an overview of the transcriptomic organization, and the relationships between sets of genes with external, biological traits. To identify key modules related to clinical traits, WGCNA was performed by using GSE26886 dataset (Fig. 3A). The power of β = 8 (scale-free R^2^ = 0.90) was selected as the soft thresholding parameter to construct a scale-free network (Fig. 3B). Similar module clustering was constructed by using dynamic hybrid cutting (threshold = 0.25). A total of 25 modules were identified (Fig. 3C). The results in Fig. 3D showed that the grey module was the highest positive module correlated to EAC (R^2^ = 0.86, P = 2e^−12^) and was highly negative correlated to BE (R^2^ = 0.86, P = 2e^−12^). Figure 3E showed gene significance for BE and EAC in grey module.

In the module-trait analysis, we intersected the trait-related genes in grey module highly associated with EAC and 131 DEGs generated from expression difference analysis, and finally extracted 27 trait-expression-related genes for the following analysis (Fig. 3F, Tables S1 and S2). These results showed that the 27 trait-expression-related genes were significantly correlated with the pathogenesis of EAC.

### Exploration of trait-expression-related genes in TCGA database

**Figure 4:**
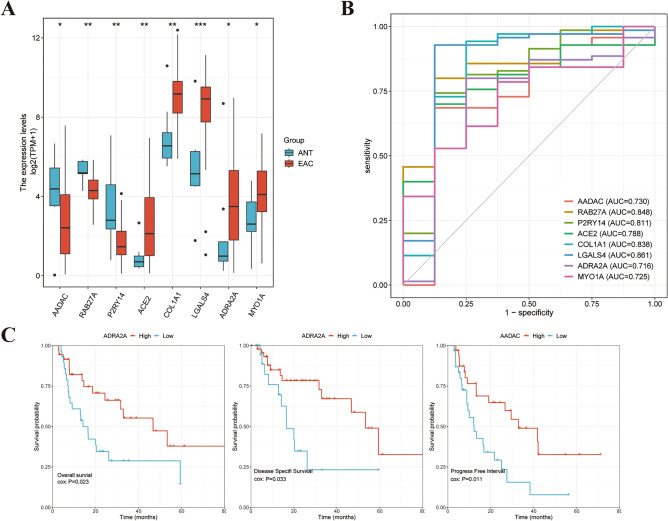
Further validation in TCGA database (*p < 0.05, **p < 0.01, ***p < 0.001). (**A**) Box plot assessing the expression of 8 genes in TCGA database; (**B**) ROC curve for the 8 genes; (**C**) Survival plots of *ADRA2A* and *AADAC* in overall survival, disease specific survival, and progress free survival.

Next, further validation and exploration were conducted among the 27 trait-expression-related genes in TCGA database. *MYO1A*, *ACE2*, *COL1A1*, *LGALS4*, and *ADRA2A* were significantly up-regulated in EAC tissue; while *AADAC*, *RAB27A*, and *P2RY14* were abnormally down-expressed in EAC tissue, which indicated that these genes were repeatable in EAC (Fig. 4A). Subsequently, ROC curves were performed to estimate the diagnostic value in EAC, and the result showed that the genes mentioned above had good diagnostic properties (Fig. 4B). Later, survival analysis was performed to explore the prognostic value of the 8 genes, and the clinical data were shown in Table 1. Low- *ADRA2A* expression was associated with poor overall survival (OS) and disease-specific survival (DSS); while low- *AADAC* expression were significantly correlated with poor progress-free interval (PFI). These results illustrated that *ADRA2A* and *AADAC* could contribute to EAC pathogenesis and progression.

**Table 1:** Clinical baseline data of EAC patients in TCGA database.

Characteristic	ADRA2A		P	AADAC		P
	Low	High		Low	High	
**Gender, n (%)**						
Female	4 (5.7%)	6 (8.6%)	0.734	4 (5.7%)	6 (8.6%)	0.734
Male	31 (44.3%)	29 (41.4%)		31 (44.3%)	29 (41.4%)	
**Age, n (%)**						
≤ 60	19 (27.1%)	5 (7.1%)	0.001	13 (18.6%)	11 (15.7%)	0.801
> 60	16 (22.9%)	30 (42.9%)		22 (31.4%)	24 (34.3%)	
**T stage, n (%)**						
T1 and T2	12 (21.4%)	13 (23.2%)	1.000	11 (19.6%)	14 (25%)	0.591
T3 and T4	14 (25%)	17 (30.4%)		17 (30.4%)	14 (25%)	
**N stage, n (%)**						
N0 and N1	24 (42.1%)	27 (47.4%)	1.000	26 (45.6%)	25 (43.9%)	1.000
N2 and N3	3 (5.3%)	3 (5.3%)		3 (5.3%)	3 (5.3%)	
**M stage, n (%)**						
M0	25 (45.5%)	26 (47.3%)	1.000	26 (47.3%)	25 (45.5%)	1.000
M1	2 (3.6%)	2 (3.6%)		2 (3.6%)	2 (3.6%)	
**Pathologic stage, n (%)**						
I and II	13 (23.2%)	17 (30.4%)	0.818	14 (25%)	16 (28.6%)	0.789
III and IV	13 (23.2%)	13 (23.2%)		14 (25%)	12 (21.4%)	

## Discussion

Currently, the pathogenesis of BE-EAC is still unclear, and the disease stratification and treatment are also limited. In the present study, we identified 27 up- and 104 down-regulated DEGs in two public datasets, and the results from GO and KEGG analysis indicated that the DEGs were highly associated with tumorigenesis. Subsequently, 27 trait-expression-related genes highly correlated with EAC were screened out by WGCNA. *MYO1A*, *ACE2*, *COL1A1*, *LGALS4*, *ADRA2A, AADAC*, *RAB27A*, and *P2RY14* were also abnormally regulated in TCGA database and represented good diagnostic properties. Surprisingly, we found that *ADRA2A* and *AADAC* were correlated with EAC prognosis.

Previous studies showed that *COL1A1*, *RAB27A*, and *P2RY14* were identified as the potential biomarker for esophageal squamous cell cancer (ESCC) and *RAB27A* associated with immune infiltration in ESCC^7,18–20^. However, there was no further experiment to verify their effects on EAC.

To the best of our knowledge, our study, for the first time, screened out 5 genes related to EAC. *MYO1A* is most highly expressed in the digestive tract, and it is associated with stomach adenocarcinoma and colon cancer^21,22^. *ACE2*, the receptor of COVID-19, is aberrantly expressed in many tumors^23^. It is reported that *LGALS4*, a β-galactoside binding protein, is correlated with prognosis in urothelial carcinoma of bladder and is also a tumor marker in serum immunoassay determination of colorectal carcinoma^24,25^. *ADRA2A* is thought to be involved in the progression of multiple cancer and can inhibit the activation of PI3K/Akt/mTOR pathway^26^. *AADAC* is a kind of serine hydrolase widely involved in the hydrolysis of drugs and associated with poor prognosis in stomach adenocarcinoma^27,28^. More future studies are needed to gain more insights into these genes.

On the one hand, our study had more strict inclusion criteria than the previous study, such as exclusion of old research, duplicated research, and patients with chemotherapy or radiation treatment, which ensured the accuracy of the results and might be enlightened for the future research or clinical guidance^29^. On the other hand, we explored some potential biomarkers that had not been reported in BE-EAC through multiple datasets. Nevertheless, our study also had several limitations. Firstly, further experiments were required to verify these results. Secondly, the lack of BE cases in TCGA database prevented us from comparing EAC and BE, which might impact the outcomes. However, there seemed to be no better way for us to compare the effects of the hub genes on diagnosis and prognosis.

In conclusion, *MYO1A*, *ACE2*, *COL1A1*, *LGALS4*, *ADRA2A, AADAC*, *RAB27A*, and *P2RY14* could be potential novel diagnostic and prognostic biomarkers in BE-EAC. In addition, *ADRA2A* and *AADAC* could contribute to EAC progression. Although further validation is still needed, we provide useful and novel information to explore the potential candidate genes for BE-EAC prognosis and therapeutic options.

## Supplementary Information

Supplementary Information.

Supplementary Table 1.

Supplementary Table 2.

## Data Availability

Publicly available datasets were analyzed in this study. This data can be found here: GEO data base, accession number: GSE26886 and GSE37200.
