# Supplementary figures and images for: Identification of potential biomarkers in Barrett’s esophagus derived esophageal adenocarcinoma
Source: Sci Rep. 2023 Feb 9;13:2345. doi: 10.1038/s41598-022-17107-0 (PMC9910260; doi:10.1038/s41598-022-17107-0)

**
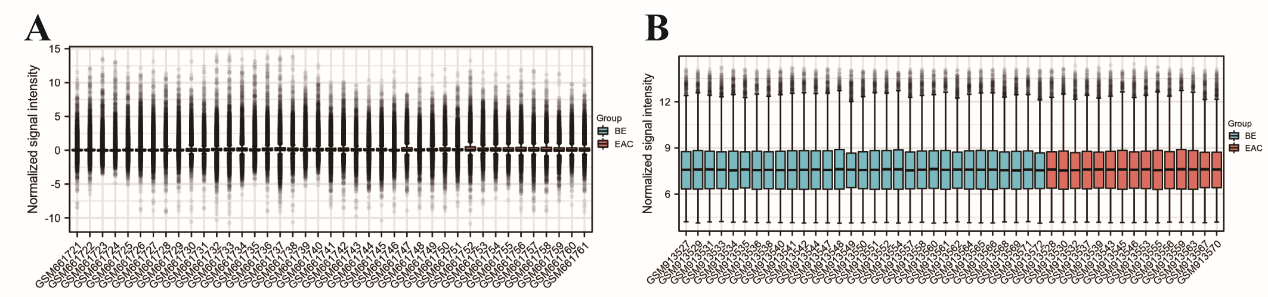
**

**Figure S1.** Batch effects correction. (A) Batch effects in GSE26886; (B) Batch effects in GSE37200.

Supplement: Supplementary file 1 — Supplementary Information. [file 41598_2022_17107_MOESM1_ESM.docx]
